# A case of successful hormone therapy for refractory hypotension following viral encephalitis: Case report

**DOI:** 10.1097/MD.0000000000034988

**Published:** 2023-10-20

**Authors:** Congcong Zhang, Jiangshan Zhang, Zhongkai Liao

**Affiliations:** a Affiliated Haikou Hospital of Xiangya Medical College, Central South University, Master of Medicine, Haikou, China; b Affiliated Haikou Hospital of Xiangya Medical College, Central South University, Master of Medicine, Haikou, China; c The Second Affiliated Hospital of Hainan Medical University, Master of Medicine, Haikou, China.

**Keywords:** corticosteroids, herpes simplex encephalitis, hypotension, intracranial infection, viral encephalitis

## Abstract

**Rationale:**

Refractory hypotension is a life-threatening condition that can result from various causes. We report a rare case of refractory hypotension following herpes simplex virus type 1 encephalitis that was successfully treated with hormone therapy.

**Patient concerns:**

The patient was a 66-year-old male who was admitted to the hospital because of fever, chills, convulsions, and impaired consciousness. He developed respiratory failure and was intubated. Cerebrospinal fluid metagenomic sequencing confirmed herpes simplex virus type 1 infection. He received piperacillin-tazobactam for anti-infection, acyclovir for antiviral therapy, and dexamethasone for anti-inflammatory therapy. He had repeated episodes of hypotension despite fluid resuscitation and vasopressor therapy.

**Diagnosis:**

The diagnosis of herpes simplex virus type 1 encephalitis complicated by refractory hypotension was based on the patient’s epidemiological history, clinical manifestations, laboratory tests, and imaging studies. Cerebrospinal fluid examination was the most important diagnostic method, which could detect viral nucleic acids. Head magnetic resonance imaging showed a large recent lesion in the right temporal-parietal and insular lobes.

**Interventions:**

The treatment of refractory hypotension mainly included anti-infection, antiviral, anti-inflammatory, and hormone therapy. Hormone therapy used methylprednisolone shock treatment until tapering withdrawal. Other treatments included fluid resuscitation, vasopressors, anticonvulsants, etc.

**Outcomes:**

The patient’s blood pressure stabilized after receiving methylprednisolone shock treatment, and his mean arterial pressure increased from 73 mm Hg to 92 mm Hg within 24 hours. Three months later, the patient’s blood pressure was normal without medication, and he had a good social and physical recovery.

**Lessons:**

This case illustrates the possible role of hormone therapy in restoring blood pressure in patients with refractory hypotension following viral encephalitis. It suggests that adrenal insufficiency or autonomic dysfunction may be involved in the pathophysiology of this condition. Further studies are needed to confirm the efficacy and safety of hormone therapy in this setting.

## 1. Introduction

Refractory hypotension is a life-threatening condition that occurs when blood pressure remains low despite adequate fluid resuscitation and vasopressor therapy.^[1]^ It can result from various causes, such as septic shock, cardiogenic shock, or adrenal insufficiency.^[1]^ Viral encephalitis, an inflammation of the brain caused by viral infection,^[2]^ is a rare cause of refractory hypotension that may involve dysfunction of the autonomic nervous system. Hormone therapy, such as steroids or vasopressin, may be effective in restoring blood pressure in these patients. In this case report, we present a patient with refractory hypotension following herpes simplex virus encephalitis who was successfully treated with steroids. To our knowledge, this is the first case of refractory hypotension following viral encephalitis that was treated with hormone therapy.

## 2. Case report

**Figure 1.**
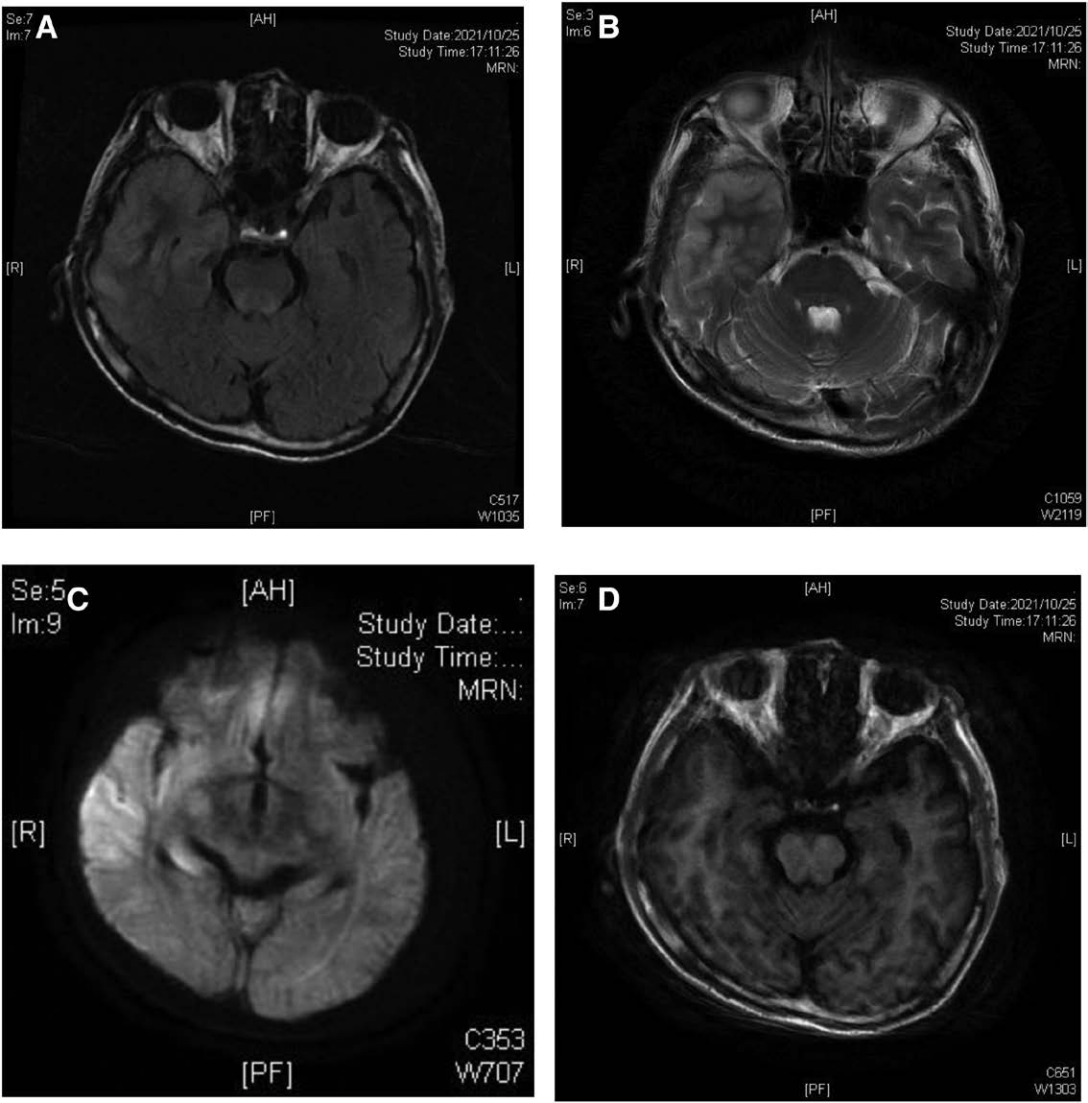
(A) T2 image, slightly hyperintense patchy lesion in the right temporal lobe. (B) FLAIR image, slightly hyperintense signal in the right temporal lobe hippocampus. (C) DWI shows high signal in the temporal and insular lobes. (D) No significant changes observed in the T1 image.

A 66-year-old male patient was admitted to our hospital on October 26, 2021, due to “slow response for 1 day, worsened for 5 hours.” The patient had been found by family members to have a slow response, reduced speech, and difficulty in speaking 1 day before the onset of the disease. The patient condition worsened 5 hours prior to admission, with a dull expression, inability to answer questions correctly, and a mild tremor in the upper and lower limbs of the left side. The patient also had a fever, but the temperature was not measured. The patient was brought to our hospital for treatment. The patient had a fall from a sitting position 4 days before the onset of the disease and underwent a head computed tomography scan at a local hospital, which showed no abnormalities. The patient had no history of infectious disease exposure or recent abdominal pain, diarrhea, nasal discharge, or cough. Physical examination upon admission: body temperature: 39°C, blood pressure: 130/70 mm Hg, clear consciousness, dull facial expression, slow response, incomplete sensory aphasia. Both pupils were equal and round, with a diameter of about 2.5 mm, uncooperative, and spontaneous movement was observed. Neck resistance was 1 transverse finger, and pathological reflexes were not elicited. Emergency head magnetic resonance imaging: recent large lesions in the right temporal-parietal lobe and insula: cerebral infarction? Multiple ischemic lesions in the brain. Cerebral white matter loosening and cerebral atrophy. Left maxillary sinus inflammation (see Fig. 1). The patient routine blood tests, coagulation function, liver and kidney function, C-reactive protein, infectious diseases, myocardial enzymes, blood lipids, tumor markers, hepatitis B 2-and-a-half, heart function, routine urinalysis, and glycosylated hemoglobin were all normal. Electrolytes: potassium: 5.50 mmol/L↑, sodium:128 mmol/L↓, chloride: 88 mmol/L↓; blood gas analyze: pH value 7.473↑, carbon dioxide partial pressure: 39.6 mm Hg, oxygen partial pressure: 85.3 mm Hg, base excess: 4.5 mmol/L↑, bicarbonate: 28.4 mmol/L↑, lactate: 1.57 mmol/L. The patient experienced left limb convulsions on the day of admission, and intravenous diazepam was administered for continuous infusion to control epileptic seizures. Blood gas analysis on the same day showed pH value: 7.524↑, oxygen partial pressure: 52.3 mm Hg↓, oxygen saturation: 89.3%↓, potassium ion: 3.44 mmol/L↓, sodium ion: 129.5 mmol/L↓, lactate: 2.29 mmol/L↑. Administer sodium supplementation, oxygen therapy via face mask, and symptomatic treatment. On October 27th, 2021, a lumbar puncture was performed, with cerebrospinal fluid (CSF) pressure at 95 mm H_2_O and clear CSF. The patient blood pressure was 104/66 mm Hg. Due to the patient recurrent high fever, piperacillin-sodium-tazobactam (imported) 4.5g q8h was empirically added for anti-infection treatment, along with acyclovir injection 0.5g q8h for antiviral treatment, and dexamethasone sodium phosphate injection 2 mL qd for anti-inflammatory treatment.^[3,4]^ Later, the patient CSF results showed CSF biochemistry: chloride: 111 mmol/L↓, glucose: 3.47 mmol/L, trace protein:1493 mg/L↑, and trace albumin: 634 mg/L↑. CSF routine: colorless, clear, red blood cells: 4–6/HP, white blood cells: 7–10/HP, Pandy test negative, white blood cell count:

159 × 10^6^/L, mononuclear white blood cells: 98%, polymorphonuclear white blood cells: 2%. CSF Cryptococcus: Cryptococcal antigen test negative, India ink staining for Cryptococcus not found; thin film test negative. CSF bacterial culture revealed no pathogens. Due to the patient persistent low blood pressure and the disappearance of limb convulsions, diazepam was discontinued after 1 day of use and replaced with oral sodium valproate sustained-release tablets 0.5g, qd for antiepileptic treatment. Later, the patient CSF second-generation sequencing results reported human herpesvirus type 1. Thus, piperacillin-sodium-tazobactam was discontinued after 4 days of use. The patient received regular antiviral treatment for 5 weeks and dexamethasone sodium phosphate injection 10 mg, qd for 10 days.^[3,4]^ During the treatment, the patient blood pressure continued to decrease, so an infusion of 32 mL saline solution and 180 mg dopamine hydrochloride injection was administered to maintain blood pressure. Fluid therapy was also given. The patient blood pressure remained low, so norepinephrine 180 mg and 41 mL saline solution were added to the dopamine infusion for increased blood pressure support, along with continued fluid therapy. The patient blood pressure was maintained during this period, so norepinephrine was discontinued on November 5th. The patient blood pressure dropped again to 95/62 mm Hg. After increasing the dose of dopamine, the patient blood pressure fluctuated between 94 to 116/58 to 78 mm Hg. Fluid therapy was continuously administered during this period. Due to the high dose of dopamine used during the continuous infusion, dopamine was discontinued on November 15th and replaced with norepinephrine 180 mg mixed with 41 mL saline solution for continuous infusion to maintain blood pressure. During the continuous infusion of norepinephrine, the patient blood pressure dropped to a low of 88/64 mm Hg. Due to the patient persistent low blood pressure, methylprednisolone sodium succinate 0.4 g was added to 250 mL saline solution on November 23rd. The dosage was reduced once every 3 days, and the patient blood pressure gradually stabilized. After discontinuing the medication, the patient blood pressure remained stable and the patient was discharged. During the period of low blood pressure, the patient remained alert and responsive, with no further convulsion symptoms. Three months after discharge, the patient daily life was not affected, self-measured blood pressure was normal, and there were no complaints of dizziness or falling symptoms.

## 3. Discussion

Currently, there are very few domestic reports on refractory hypotension following herpes simplex encephalitis. There have been several individual cases of hypotension due to autoimmune encephalitis reported in foreign journals. In these cases, patients developed refractory hypotension after encephalitis, which significantly prolonged hospitalization time and increased hospitalization times. Kenta Orimo^[5]^ and others have reported a case of Anti-leucine-rich glioma inactivated 1-associated autoimmune encephalitis in which the patient developed severe orthostatic hypotension 5 years after clinical recovery. The patient orthostatic hypotension quickly improved after immunoglobulin pulse therapy, but severe orthostatic hypotension recurred 2 months later, and gradually recovered after another round of immunoglobulin pulse therapy. Since the interval between the encephalitis and orthostatic hypotension in this case was too long, the relationship between the two is not very certain. Takenobu Murakarni found a case of anti-N-methyl-D-aspartate receptor encephalitis that also developed orthostatic hypotension during treatment.^[6]^ After receiving high-dose steroid pulse therapy and immunoglobulin pulse therapy, the patient took quixotic dopamine capsules long-term to improve hypotension. Both of the above-reported cases had lesions involving the brainstem. Kong Hanxin^[7]^ and others reported in 2020 a case of anti-gamma-aminobutyric acid antibody-associated autoimmune encephalitis that developed refractory hypotension during treatment, with lesions involving the hippocampus area. After the diagnosis was confirmed, high-dose steroid pulse therapy was administered. When the dose was reduced to oral steroids, the blood pressure dropped. The subsequent immunoglobulin pulse therapy had a poor effect, but after the addition of oral steroids, the blood pressure gradually stabilized. In several cases, patients experienced difficulties in correcting hypotension, which significantly prolonged hospital stays and increased the number of hospitalizations.

Herpes simplex encephalitis is a common lethal intracranial infection, with typical neuroimaging findings of high signal foci in the bilateral temporal or frontal lobes.^[8]^ However, the mechanism and treatment of refractory hypotension after herpes simplex encephalitis are not clear. In this article, we report a case of refractory hypotension after herpes simplex encephalitis, which returned to normal blood pressure after low-dose steroid pulse therapy. Autoimmune encephalitis is a group of autoimmune antibody-mediated inflammatory diseases of the nervous system with diverse clinical manifestations, including cognitive impairment, psychiatric and behavioral abnormalities, seizures, movement disorders, and autonomic dysfunction.^[9]^ The relationship between autoimmune encephalitis and hypotension is not clear, but several reports have shown that some patients with autoimmune encephalitis developed hypotension or orthostatic hypotension during treatment.^[5–7,10,11]^ Among these patients, some had lesions involving the brainstem,^[5,7,11]^ some had lesions in the hippocampal region, and some had specific autoantibodies.^[5-7,11]^ These factors may be related to autonomic dysfunction or the influence of the central nervous system on peripheral vascular regulation. In our reported case, whether there is a possibility of autoimmune encephalitis following simple herpes virus encephalitis was considered, but due to the patient financial reasons, they refused to undergo further blood and CSF tests to detect related antibodies for autoimmune encephalitis. The patient had a history of falling after standing up 4 days before being admitted to our hospital. Whether orthostatic hypotension had already existed before the change in mental status and whether orthostatic hypotension is a unique manifestation of intracranial infection or autoimmune encephalitis are questions that need to be verified from more cases. We found that low-dose steroid pulse therapy had a significant improvement effect on patients’ hypotension. This may be related to multiple mechanisms such as the suppression of the autoimmune response by steroids, the protection of vascular endothelial function, and the activation of the vasoconstrictor system.^[10,12,13]^ Some patients with autoimmune encephalitis also showed improvement in hypotension or orthostatic hypotension after receiving high-dose steroid pulse therapy,^[6,7,11]^ while we used low-dose steroid pulse therapy with positive results. However, there are currently no unified guidelines or consensus on the use and dosage of steroids in the treatment of post-encephalitis hypotension. The specific usage methods and dosages will hopefully provide us with better guidance as more cases accumulate.

Treatment-resistant hypotension can significantly prolong hospital stay and increase the number of hospital admissions due to recurrent episodes of hypotension, which can lower the quality of life for patients. We speculate that these hypotensive episodes may be caused by dysfunction of the autonomic nervous system. Due to the limited number of case reports, the specific mechanism underlying hypotension after encephalitis remains unclear, and whether there are specific antibodies involved in the pathogenesis is still unknown. We hope that with the gradual increase of cases and more research into the mechanism of hypotension, we can uncover more clues.

## Author contributions

**Data curation:** Jiangshan Zhang.

**Investigation:** Jiangshan Zhang.

**Writing – original draft:** Congcong Zhang.

**Writing – review & editing:** Zhongkai Liao.

## References

[1] Garg RK Paliwal VK Gupta A. Encephalopathy in patients with COVID-19: a review. J Med Virol. 2021;93:206–22. 32558956 10.1002/jmv.26207

[2] Smith LL Branson BW. REFRACTORY HYPOTENSION-diagnosis and management in surgical patients. Calif Med. 1961;95:150–5. 18732435 PMC1574467

[3] Piret J Boivin G. Immunomodulatory strategies in herpes simplex virus encephalitis. Clin Microbiol Rev. 2020;33:e00105–19. 32051176 10.1128/CMR.00105-19 PMC7018500

[4] Smuts I Lamb GV. Viral infections of the central nervous system. In: Green RJ Lamb GV Smuts I, eds. Viral Infections in Children. Volume II. Cham, Switzerland: Springer International Publishing; 2017:83–123.

[5] Orimo K Iwata NK Kawai M. Anti-LGI1 encephalitis developing immunoglobulin responsive orthostatic hypotension after remission. Intern Med. 2021;60:3021–4. 33055478 10.2169/internalmedicine.5359-20 PMC8502651

[6] Hanxin K Xiaomin W Hao Y. Case report: refractory hypotension of GABA B receptor autoimmune encephalitis. Front Neurol. 2020;11:571382. 33335508 10.3389/fneur.2020.571382 PMC7736632

[7] Solomon T Michael BD Smith PE. Management of suspected viral encephalitis in adults--Association of British Neurologists and British Infection Association National Guidelines. J Infect. 2012;64:347–73. 22120595 10.1016/j.jinf.2011.11.014

[8] Graus F Titulaer MJ Balu R. A clinical approach to diagnosis of autoimmune encephalitis. Lancet Neurol. 2016;15:391–404. 26906964 10.1016/S1474-4422(15)00401-9 PMC5066574

[9] Annane D Pastores SM Rochwerg B. Guidelines for the diagnosis and management of critical illness-related corticosteroid insufficiency (CIRCI) in critically ill patients (Part I): Society of Critical Care Medicine (SCCM) and European Society of Intensive Care Medicine (ESICM) 2017. Intensive Care Med. 2017;43:1751–63. 28940011 10.1007/s00134-017-4919-5

[10] Murakami T Nakatani-Enomoto S Enomoto H. A unique shape of brainstem lesion that caused orthostatic hypotension in Anti-NMDAR Encephalitis. Intern Med. 2019;58:2861–4. 31178512 10.2169/internalmedicine.2805-19 PMC6815904

[11] Seo JH Lee YJ Lee KH. Autoimmune encephalitis and epilepsy: evolving definition and clinical spectrum. Clin Exp Pediatr. 2020;63:291–300. 31431603 10.3345/kjp.2019.00598 PMC7402981

[12] Annane D Bellissant E Bollaert PE. Corticosteroids for treating sepsis in children and adults. Cochrane Database Syst Rev. 2019;2019:CD002243. 10.1002/14651858.CD002243.pub4 PMC6953403 31808551

[13] Marik PE Pastores SM Annane D. Recommendations for the diagnosis and management of corticosteroid insufficiency in critically ill adult patients: consensus statements from an international task force by the American College of Critical Care Medicine. Crit Care Med. 2008;36:1937–49. 18496365 10.1097/CCM.0b013e31817603ba

